# Access to health care for older people with intellectual disability: a modelling study to explore the cost-effectiveness of health checks

**DOI:** 10.1186/s12889-019-6912-0

**Published:** 2019-06-07

**Authors:** Annette Bauer, Laurence Taggart, Jill Rasmussen, Chris Hatton, Lesley Owen, Martin Knapp

**Affiliations:** 1 Personal Social Services Research Unit, London School of Economics and Political Science, Houghton Street, London, England WC2A 2AE UK; 2 Institute of Nursing & Health Research, Ulster University, N Ireland, Newtownabbey, BT37 0QB UK; 3 Royal College of General Practitioners (RCGP), 30 Euston Square, London, England NW1 2FB UK; 4 Centre for Disability Research, Division of Health Research, Lancaster University, Lancaster, England LA1 4YG UK; 5 National Institute for Health and Care Excellence, 10 Spring Gardens, London, England SW1A 2BU UK

**Keywords:** Health checks, Assessment, Prevention, Early diagnosis, Ageing, Intellectual disability, Health inequalities, Cost-effectiveness, Decision-analytic modelling

## Abstract

**Background:**

Whilst people with intellectual disability grow older, evidence has emerged internationally about the largely unmet health needs of this specific ageing population. Health checks have been implemented in some countries to address those health inequalities. Evaluations have focused on measuring process outcomes due to challenges measuring quality of life outcomes. In addition, the cost-effectiveness is currently unknown. As part of a national guideline for this population we sought to explore the likely cost-effectiveness of annual health checks in England.

**Methods:**

Decision-analytical Markov modelling was used to estimate the cost-effectiveness of a strategy, in which health checks were provided for older people with intellectual disability, when compared with standard care. The approach we took was explorative. Individual models were developed for a selected range of health conditions, which had an expected high economic impact and for which sufficient evidence was available for the modelling. In each of the models, hypothetical cohorts were followed from 40 yrs. of age until death. The outcome measure was cost per quality-adjusted life-year (QALY) gained. Incremental cost-effectiveness ratios (ICER) were calculated. Costs were assessed from a health provider perspective and expressed in 2016 GBP. Costs and QALYs were discounted at 3.5%. We carried out probabilistic sensitivity analysis. Data from published studies as well as expert opinion informed parameters.

**Results:**

Health checks led to a mean QALY gain of 0.074 (95% CI 0.072 to 0.119); and mean incremental costs of £4787 (CI 95% 4773 to 5017). For a threshold of £30,000 per QALY, health checks were not cost-effective (mean ICER £85,632; 95% CI 82,762 to 131,944). Costs of intervention needed to reduce from £258 to under £100 per year in order for health checks to be cost-effective.

**Conclusion:**

Whilst findings need to be considered with caution as the model was exploratory in that it was based on assumptions to overcome evidence gaps, they suggest that the way health systems deliver care for vulnerable populations might need to be re-examined. The work was carried out as part of a national guideline and informed recommendations about system changes to achieve more equal health care provisions.

**Electronic supplementary material:**

The online version of this article (10.1186/s12889-019-6912-0) contains supplementary material, which is available to authorized users.

## Background

Older people with intellectual disability (ID) have more health conditions than people of a similar age in the general population, but many of these go undetected and untreated [1]. The higher morbidity has been explained by a combination of genetic and lifestyle factors, as well broader social determinants of health, which means that people are much more likely to experience pervasive disadvantage [1–4]; this includes active discrimination and other barriers people with ID face when accessing standard health services [5]. Whilst those factors play a role for all people with ID independently of their age, they accumulate over a person’s lifetime and have a greater impact on quality of life as people age [5–9]. Health conditions typically associated with ageing in the general population often occur at a much earlier age, with higher prevalence and in combination [9–14]. There are substantial challenges of identifying health conditions early in this population due to ‘diagnostic overshadowing’, i.e. where physical and mental health symptoms are not only misattributed to the ID but also to age-related changes [5, 15]. The consequences are delayed diagnosis and treatment [5, 7, 15–17]. As a result, their life expectancy at birth – although increasing – is 20 years lower than for people without ID [18–20].

Health checks for people with ID have been introduced in countries such as the United Kingdom, Canada and Australia to improve detection, treatment and prevention of new health conditions in this population [21, 22]. In England, health checks were introduced in 2008 in the form of a national scheme which incentivises general practices to offer checks to people registered as having an ID each year (which is why they are typically referred to as ‘annual health checks’) [23]. Staff from practices which opt into the scheme are required to undergo specialist training. This includes the use of templates such as the Cardiff Health Check, and more recently the National Electronic Health Check; the latter incorporates a wide range of questions about health conditions including ageing-related disorders, bowel and breast cancer screening and tests for osteoporosis. It also includes a section on mental health [24].

Evaluations of the national scheme found that annual health checks (AHCs) led to the identification of unmet health needs and unrecognised life-threatening conditions [21, 23]. However, those ‘effectiveness’ evaluations have focused on short-term process measures, such as the number of health checks attended, health assessments and investigations done and common health conditions diagnosed. One would expect that AHCs have an impact on health-related quality as well as on quantity of life because health conditions are identified and treated earlier on, or sometimes even prevented. However, robust evidence in this regard is still lacking. In addition, there is a gap in knowledge about the cost-effectiveness of AHCs [23].

The aim of this explorative economic study was to estimate impacts on long-term quality and quantity of life and costs of the AHC scheme for older people with ID in England. The work was carried out to inform a national guideline on this topic for the National Institute for Health and Care Excellence [25].

## Methods

### Procedure

We carried out an explorative cost-utility analysis comparing a strategy in which older people with ID were offered health checks every year in general practice (primary care) versus a strategy in which they received standard primary care. The first referred to care provided in general practices as part of the AHC scheme, whilst standard care referred to care provided by general practices not part of such scheme. We hypothesized that earlier identification of health conditions due to regular check-ups would lead to health improvements as well as potential reductions in costs due to fewer treatments for health conditions at a more severe stage. The analysis was explorative in nature and it was not the aim of the analysis to include all possible economic consequences of AHCs. Instead, the model focused on those health conditions that were covered by current checklists used in primary care and most important in terms of their (expected) economic impact. Decision-analytical Markov models were constructed to estimate lifetime quality-adjusted life years (QALYs) and costs from a National Health Service (NHS) perspective in 2016 prices, discounted at a standard annual rate of 3·5% [26]. For each of a selected range of health conditions a separate Markov model was developed, generating the present values of lifetime cost and QALY gain linked to earlier identification or treatment of this particular health condition. We then aggregated the results of all single models in terms of their present values of lifetime costs and QALY gains. Double counting of overlapping health conditions was avoided by adjusting prevalence rates, or by only including those conditions that were expected to occur first. The net present value of the costs of yearly health checks was then added to those aggregated costs to derive a final incremental cost-effectiveness ratio (ICER). The modelling considered a hypothetical population aged 40 years (although for some health conditions older starting ages were applied), who were followed until death. The models were constructed using Microsoft Excel and applied half cycle corrections, probabilistic sensitivity analysis and Monte Carlo simulation to assess the impact of changes in parameters on the ICER.

The method is explained by describing the general approach that was applied across all models; where there was a deviation from this approach (for a particular health condition), this is highlighted. A technical report is available on NICE’s website, which describes each single model for each health condition [27].

Sources from which data were taken to inform the modelling are referred to and described under respective sub sections. Data referred to population or clinical samples. For example, a large Irish cohort study was the primary source for probabilities that people developed health conditions. Probabilities for the identification of health conditions stemmed from data from a large primary care database in England. We used data from clinical studies to model the likely consequences of an earlier versus later identification of health conditions. Details of how the literature was searched for this study are provided in Additional file 1.

**Fig. 1:**
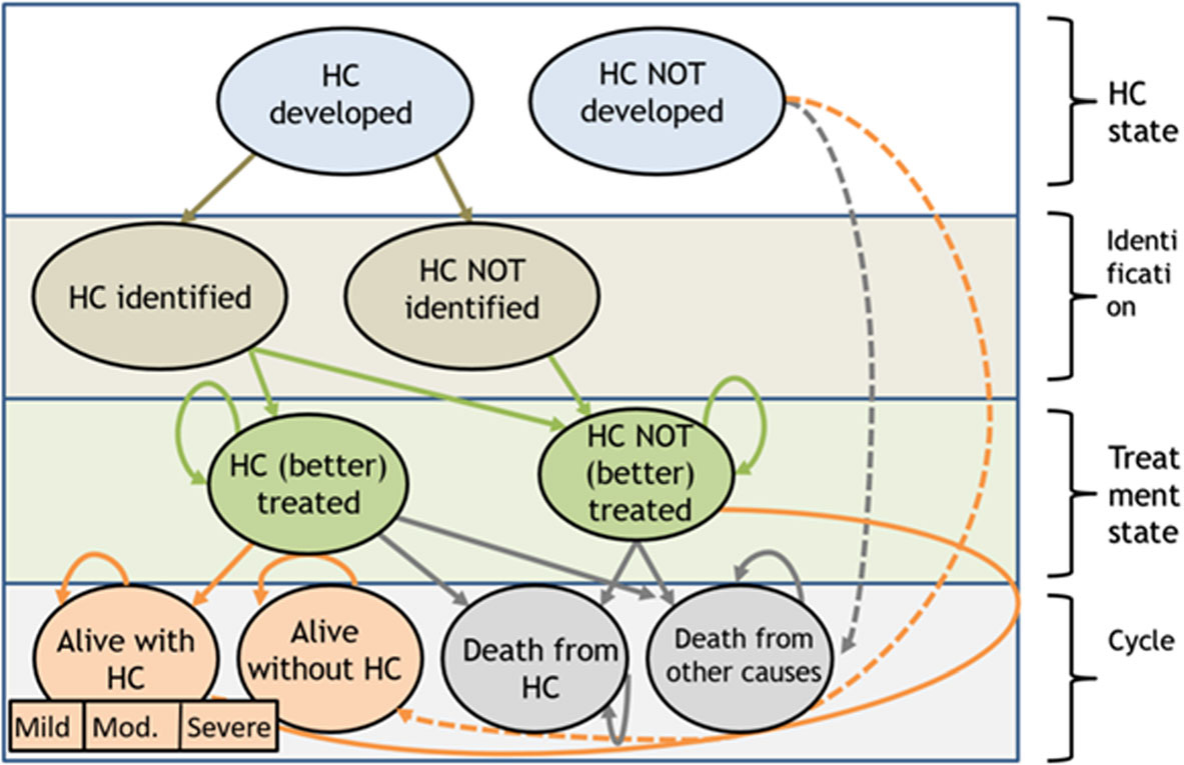
Markov modelling, simplified transition-state-diagram (HC=Health condition)

Figure 1 illustrates the key health states in our models and possible transitions between them during each cycle. Each year a person could develop a health condition, be identified with a health condition (or not), get treatment (or not), survive, or die (either because of the health condition or because of other causes). Of those people alive, they could live throughout each yearly cycle with or without a health condition, which was either treated or not. For some health conditions (high blood pressure, diabetes), a distinction was made whether a person’s health condition was managed well (due to regular check-ups provided during AHCs) or just treated normally. Whether a person was identified with a health condition and received treatment for it (or whether the health condition was managed more closely rather than just treated normally) could influence the disease progression and yearly probability of death from the condition (if indicated by evidence). A quality of life weight (in the form of health utilities) and a cost were assigned to each event state (with death set equal to zero). We ran the models and calculated total costs and QALYs based on the time each person spent in each health state.

### Health conditions: selection, incidence and progression

A selected number of ageing-relevant health conditions were included in our modelling: osteoporosis; breast cancer; bowel cancer; cataract; glaucoma; hearing problems; diabetes; hypertension. Coronary heart disease (CHD) and stroke were modeled as consequences of diabetes or hypertension; hip fracture was modeled as a consequence of osteoporosis. Not included due to a lack of economic evidence or because of expected low economic impact were: cervical cancer screening (smear test); prostate cancer; lung cancer; body mass index, cholesterol, weight; thyroid problems; arthritis; chronic obstructive pulmonary disease (COPD) and asthma; epilepsy; immunization status; mental health and dementia. The rationale for why health conditions were included or excluded is presented in Table 1 (and a more detailed explanation is provided in Additional file 1: Table S1).

**Table 1:** Rational for including or excluding health conditions

Health condition	Included/ excluded	Rationale
Arthritis	Excluded	Despite a high prevalence, the expected impact on costs and outcomes was likely to be low or medium due to uncertainties around optimal identification and management; in addition there was no consistent evidence of whether AHC would improve the identification or management of arthritis.
High blood pressure (hypertension)	Included	The expected impact on costs and outcomes was high; robust (cost-) effectiveness evidence for blood pressure management was available; evidence was also available that showed that AHC led to improved identification and management of high blood pressure.
Body mass index, weight, cholesterol	Excluded	Overall, there was only limited evidence that AHC was able to influence those health promotion outcomes.
Bowel cancer screening	Included	The expected impact on costs and outcomes was high because of a high prevalence of the condition, the availability of a national screening programme, and the availability of (cost-) effective treatment; although uptake has not been considered in the evaluations of AHCs there is evidence that additional information provided by general practitioners increases uptake.
Breast cancer (screening via mammogram)	Included	The expected impact on costs and outcomes was high because of the high prevalence of the condition, the availability of a national screening programme, and of cost-effective treatment; although uptake has not been considered in the evaluations of AHCs there is evidence that additional information provided by general practitioners increases uptake.
Cataract	Included	The expected impact on costs and outcomes was high because of the high prevalence, availability of (cost-) effective treatment, and strong evidence that AHCs led to an increase in eye tests.
Cervical cancer screening	Excluded	The expected impact on costs and outcomes was low because of the low prevalence in this population.
COPD and asthma	Excluded	Evidence was insufficient: the prevalence of asthma was not well established for this population; there was no evidence that AHC would lead to changes in the identification or management of COPD or asthma.
Dementia	Excluded	Evidence was insufficient; in particular it was not clear whether dementia was currently checked in AHCs, and whether AHCs led to better identification.
Epilepsy	Excluded	Evidence was insufficient; in particular there was not enough robust evidence of cost-effective treatment.
Heart disease	Included (indirectly)	Heart disease was modelled as a consequence of hypertension and diabetes, which were strong predictors of heart disease. Heart disease was not modelled separately to avoid double of counting economic consequences.
Hearing impairment	Included	The expected impact on costs and outcomes was high due to the high prevalence and high impact for this population. There was robust evidence that AHC led to an increase in hearing tests; (cost-) effective treatment was available.
Glaucoma	Included	The expected impact on costs and outcomes was high; the impairment linked to glaucoma was and there was strong evidence that AHC led to more eye tests being carried out; (cost-) effective treatment was available.
Hip fracture	Included (indirectly)	This was modelled as a consequence of osteoporosis, which was a strong predictor fracture. Hip fracture was not modelled separately to avoid double counting of economic consequences.
Immunisation status	Excluded	The expected impact on costs and outcomes was low; checking for immunisation status is part of another incentivised scheme in primary care. This suggested a more limited role of AHCs in further improving uptake.
Lung cancer/ smoking	Excluded	The expected impact on costs and outcomes was low due to lack of evidence of cost-effective treatment options that would be influenced by an earlier identification; also lack of robust evidence whether identification improved through AHC.
Mental health	Excluded	Evidence was insufficient; whilst prevalence data were available, there was no evidence about whether AHC led to a better identification of mental health problems; there was also a lack of evidence regarding (cost-) effective treatment options for this population.
Osteoporosis (screening)	Included	The expected impact on costs and outcomes was high due the high prevalence and the availability of screening tools that led to an increase in the identification of osteoporosis and reduction in (costly) fractures. Screening for osteoporosis is covered by the new AHC tool in England.
Prostate cancer	Excluded	Evidence was insufficient; prevalence data were not available and there was no robust evidence about (cost-) effective treatment options and whether AHCs led to increase in identification or improved management of the condition.
Stroke	Included (indirectly)	This was modelled as a consequence of hypertension and diabetes, which were strong predictors of stroke. Stroke was not modelled separately to avoid double counting economic consequences.
Thyroid problems	Excluded	There is an overall lack of evidence suggesting that expected impact of identification or monitoring through annual health checks is likely to have a large impact on costs or health outcomes.

Each health condition was modelled separately. For conditions known to interact with each other substantially (diabetes and hypertension), incidence rates were adjusted to avoid double-counting of costs and outcomes. For the same reason, conditions (such as heart diseases, stroke or hip fracture) typically preceded by conditions already covered in the modelling (hypertension, diabetes or osteoporosis in these cases) were not modelled separately. The model focused on the new occurrence (incidence) of conditions and did not consider conditions that existed before the person reached the age of 40 years (or respective starting ages). Data on the yearly incidence and progression of conditions were derived from the Intellectual Disabilities Supplement to the Irish Longitudinal Study on Aging (IDS-TILDA) [17], with the exception of hearing problems, for which data were taken from two other sources because they were not available from IDS-TILDA [28, 29]. Parameters, their values, sources and descriptions are shown in Table 2.

**Table 2:** Parameter values (deterministic, in ranges) for modelling: Yearly probabilities for developing health conditions, and cohort starting ages

	Data	Source and details
Yearly probabilities for developing health conditions		
Hypertension, diabetes excluded		
40 to 49 years	0·35 to 1·14%	Derived from 3 years incidence data from IDS-TILDA by McCarron and colleagues [17]
50 to 64 years	1·56 to 2·28%	As above
65 years+	2·39 to 4·76%	As above
Stroke		
40 to 49 years	0 to 1·11%	As above
50 to 64 years	0·23 to 1·13%	As above
65 years+	0·44 to 2·85%	As above
Coronary heart disease (CHD)		
40 to 49 years	0	As above
50 to 64 years	0·07 to 0·91%	As above
65 years+	0·44 to 2·82%	As above
Diabetes		
40 to 49 years	0 to 1·11%	As above
50 to 64 years	0·03 to 1·32%	As above
65 years +	0·07 to 1·83%	As above
Obesity		
Proportion with obesity (all ages)	33·5%	As above
Bowel cancer		
50 to 64 years	0·24 to 1.28%	Derived from 3 years incidence data for all types of cancer from IDS-TILDA by McCarron and colleagues [17] applied to proportion of bowel cancer among all types of cancer from Cancer Research UK [30]
65 years+	0·3 to 2·49%	As above
Breast cancer		
50 to 64 years	0 to 0·16%	Derived from 3 years incidence data for all types of cancer from IDS-TILDA by McCarron and colleagues [17] applied to proportion of breast cancer among all types of cancer from Cancer Research UK [30]
65 years +	0·04 to 0·19%	As above
Osteoporosis		
50 to 64 years	2·8 to 5·9%	Derived from 3 years incidence data from IDS-TILDA by McCarron and colleagues [17]
65 years +	4·8 to 11·2%	As above
HIP fracture		
50 to 64 years	0·07 to 0.55%	As above
65 years +	0·08 to 1·11%	As above
Cataract		
40 to 49 years	0·9 to 3%	As above
50 to 64 years	1·32 to 3·2%	As above
65 years +	1·04 to 4·09%	As above
Glaucoma		
40 to 49 years	0 to 1.2%	As above
50 to 64 years	0·14 to 1·1%	As above
65 years +	0 to 0·15%	As above
Hearing problems		
All ages	2 to 13·4%	Carvill [28] and Kerr and colleagues [29]
Cohort starting ages (if different from 40 years), years		
Bowel cancer	60	Starting age of national screening programme
Breast cancer	50	As above
Osteoporosis	50	Age when prevalence strongly increases according to data from IDS-TILDA by McCarron and colleagues [17]

### Populations: starting ages, gender and mortality

Generally, a starting age of the cohorts of 40 years was used because this was consistent with that used by IDS-TILDA (and was considered appropriate by our experts due to the earlier onset of age-related conditions). However, for certain health conditions for which AHCs led to changes in screening uptake (breast and bowel cancer) or for which incidence rates increased strongly at a later age (osteoporosis), different starting ages were used according to the age from when screening was offered (or from which prevalence strongly increased) (Table 2). We calculated yearly age-specific transition probabilities from alive to dead using the National Life Tables for England [30]. Adjustments were made reflecting the three times higher mortality rate for people with ID [6]. For health conditions which could end in death (breast cancer, bowel cancer, diabetes, hypertension), additional calculations were carried out to derive yearly probabilities of death from those causes using national data sources [31–34].

### Uncertainty

The impact of uncertainty around parameter values on the ICER was examined using probabilistic sensitivity analysis (PSA). In PSA, the full value range rather than a single value is considered for each parameter. This was done by determining the distributions that a value could take for each parameter and then running a large number (1000) of Monte Carlo simulations, which produced the results of different combinations of random draws. The choice of distributions followed the approach suggested by Sculpher [35]. In addition, one-way sensitivity analysis was applied for values that were particularly uncertain.

### Costs of the scheme

The costs of health checks were estimated in consultation with experts from the Committee. They were asked to estimate the resource inputs required for delivering AHC according to best practice. Unit costs were then attached from national sources [36]. The relevant parameters, values, sources and details are shown in Table 3. Based on yearly costs we calculated the present value of total costs over a person’s lifetime starting from 40 years of age.

**Table 3:** Parameter values (deterministic, in ranges) for modelling: Cost inputs

	Data	Source and details
Cost inputs for annual health checks		
General practice doctor	£72	PSSRU [36]; refers to 20 min of general practice doctor time with unit cost per hour of face-to-face time of £216 (includes all administrative, preparation and follow-up costs, cost of home visits)
General practice nurse	£43	PSSRU [36]; refers to 1 h of general practice nurse time with unit cost per hour of £43
Support worker	£136	Expert view; refers to 8 h of support worker time with unit cost per hour of face-to-face time of £17
Social worker	£7	PSSRU [36]; refers to 5 min of social worker time with unit cost per hour of client-related time of £79
Cost inputs for modelling hypertension		
Diagnosis	£29 to £89	Lovibond and colleagues [37]
Hypertension management	£34 to £102	As above
Treating stroke (initially)	£5633 to £16,901	As above
Subsequent treatment of stroke	£619 to £1856	As above
Treating coronary heart disease (initially)	£1854 to £5561	As above; includes costs of heart failure (£2929), angina (£3273), heart attack = myocardial infarction, MI (£5455); weighted by their prevalence proportions from IDS-TILA by McCarron and colleagues [17] in relation to all coronary heart disease conditions: heart failure (51%), angina (21%), MI (28%)
Subsequent treatment of coronary heart disease	£143 to £428	As above; includes costs of heart failure (£311), angina (£187), heart attack = myocardial infarction, MI (£312); weighted by their prevalence proportions from IDS-TILA by McCarron and colleagues [17] in relation to all coronary heart disease conditions: heart failure (51%), angina (21%), MI (28%)
Cost inputs for modelling diabetes		
∆ Controlled vs. uncontrolled glucose, non-overweight patients	-£618 to £2877	Clarke and colleagues [38]; refers to present of total lifetimes costs; for non-overweight patients treated with insulin
∆ Controlled vs. uncontrolled glucose, overweight patients	-£5486 to £2875	As above; refers to present of total lifetimes costs; for overweight patients treated with metformin
Cost inputs for modelling bowel cancer (screening)		
FOBT tests	£16 to £19	Tappenden and colleagues [39]; refers to 2 FOBT tests (in case first is not returned)
Colonoscopy	£469 to £573	National Schedule for Reference Costs 2015–16; refers to diagnostic colonoscopy [40]
Removing adenoma	£122 to £149	As above
Admittance for bleeding	£712 to £870	As above
Bowel cancer treatment detected through screening	£5971 to £7298	Cancer Research UK [30]
Bowel cancer treatment clinically detected	£7782 to £9511	As above
Cost inputs for modelling breast cancer (screening)		
Mammogram per woman invited for screening	£14 to £36	Pharoa and colleagues [31]
Treating over-diagnosis	£2047 to £2501	As above
∆ Treatment, early vs. late stage cancer, under 65 years (lifetime)	-£11,739 to -£14,347	Laudicella and colleagues [41]
∆ Treatment, early vs. late stage cancer, over 65 years (lifetime)	-£6404 to -£7827	As above
Cost inputs for modelling osteoporosis		
DAX scan and General Practitioner (GP) consultation	£113 to £137	NICE [41, 43]
Anti-osteoporotic medication (per year)	£54 to £334	NICE [41, 43]; refers to the most commonly prescribed drugs: alendronate, etidronate risedrinate, raloxifene and strontium ranelate; and recommended doses
Treating hip replacement (HIP) fracture, 1st year	£14,481 to £14,800	Leal and colleagues [44]
Treating HIP fracture, 2nd year	£2160 to £2272	As above
Cost inputs for modelling cataract		
Initial optometrist test	£21	DH [45]
Optometrist diagnosis test	£92 to £472	Burr and colleagues [46]
Cataract surgery (lifetime)	£1218 to £9211	Frampton and colleagues [47]
Cost inputs for modelling glaucoma		
Initial optometrist test	£21	DH [45]
Optometrist diagnosis test	£92 to £472	Burr and colleagues [46]
Treating mild glaucoma (per year)	£259 to £777	As above
Treating moderate glaucoma (per year)	£325 to £875	As above
Treating severe glaucoma (per year)	£232 to £695	As above
Treating visual impairment (per year)	£721 to £927	As above
Cost inputs for modelling hearing problems		
Ear wax removal	£36 to £44	Clegg and colleagues [48]; refers to primary care
Hearing specialist assessment	£46 to £68	NHS National Tariff 2017 to 2019 [49]
Hearing aid assessment	£48 to £58	As above
Hearing aid, initial	£268 to £370	As above
Hearing aid, follow on care	£23 to £28	As above

### Cost consequences

Unit costs were attached to the different states. This included the cost of participating in (additional) screening tests and other procedures for the diagnosis of health conditions or (immediate) follow-on treatment; in the case of breast cancer this included the cost of over-diagnosis due to additional screening procedures. Costs also included those for yearly treatment. Data for the unit cost of procedures or tests and cost for treating conditions were taken from recent national sources or economic evaluations [31, 40–49, 36–39]. In some instances [38, 41], cost estimates referred to present values of differences in life-time costs - in which case we directly assigned those cost differences to the additional risk that someone would be identified with the health condition (breast cancer) or would get monitored annually in the AHC group (diabetes). Parameters, value, sources and details are shown in Table 3.

### Effectiveness

Effectiveness of AHCs referred to increased access to standard treatment as a result of changes in identification of health conditions or – in the case of diabetes and hypertension - to better management of those conditions due to regular check-ups. Thus, in each of the models, people in the AHC and standard care groups had different probabilities that their health conditions would be identified, or that their condition would be well managed (people receiving AHCs had a greater probability). For the two cancers covered in the modelling (bowel and breast cancers), this included data on increased screening uptake due to reminders provided during AHCs. Earlier identification and increased access to treatment (or better management of health conditions) were modelled reflecting – where indicated by evidence – lower probabilities of disease progression, progression into more severe disease, and of death.

Data on uptakes of AHC, identification rates for health conditions and better management of health conditions were taken from a large national evaluation [23], as well as from international evaluations of AHCs [50, 51], and from expert views. Data on access to treatments (including adherence to treatment) for those identified with a health condition were taken from national statistics and economic evaluations [31, 51–63]. Parameters, their values, sources and descriptions are shown in Table 4.

**Table 4:** Parameter values (deterministic, in ranges) for modelling: Inputs for effectiveness (including data on identification, screening uptake, progression, further investigation and treatment)

	Data	Source and details
Effectiveness of annual health checks in terms of: identification; uptake of national screening; management of conditions (in probabilities, annual health check vs. standard care group)		
Hypertension identified and managed	85 to 95·3% vs. 71·4 to 87·8%	Buszewicz and colleagues [23]
Participation in bowel cancer screening (FOBT), difference between AHC and standard care, in percentage points	4·1 to 7·8%	Hewitson and colleagues [52] and expert views
Participation in breast cancer screening (mammogram)	54·3 to 59·3% vs. 47 to 52%	Derived from IDS-TILDA by McCarron and colleagues [17], Gardner and colleagues [53] and expert views
Diabetes managed (identification found similar in both groups)	69·9% (SD 34·2) vs. 56.8% (SD 29·4)	Cooper and colleagues [16]; refers to proportion of people whose health monitoring needs are met
Osteoporosis investigated	85 to 95% vs. 66·2 to 86·8%	Derived from Lennox and colleagues [49] and expert views
Person with eye problem (cataract or glaucoma) is referred to eye exam	90% vs. 58.9% (SD 0·24)	Derived from Buszewicz and colleagues [23] and expert views
Person with hearing problems is referred to hearing assessment	90% vs. 27·6 to 33%	As above
Effectiveness of: identification; uptake of national screening; management of conditions		
Relative Risk (RR) in stroke, CHD and death, managed versus unmanaged hypertension		
Stroke		
40 to 59 years	0·61 to 0·65	Moran and colleagues [52]
60 years +	0·66 to 0·71	As above
CHD		
40 to 59 years	0·72 to 0·74	As above
60 years +	0·74 to 0·78	As above
Death		
40 to 59 years+	0·83 to 0·89	As above
60 years +	0·91 to 0·92	As above
Increased risk of stroke in people with hypertension	3 to 5	Straus and colleagues [55]
Increased risk of CHD in people with hypertension	2 to 3	Padwal and colleagues [54]
Absolute risk reductions (in percentage points) in death for people participating in bowel screening	1·01%	Scholefield and colleagues [57]
Progression probabilities for glaucoma, treatment vs. not in treatment		
Progression from mild to moderate	22% vs. 25%	Burr and colleagues [44]
Progression from moderate to severe	7% vs. 11%	As above
Progression from severe to visual impairment	6% vs. 10%	As above
Relative risk reduction of death from breast cancer for women invited for mammography	0·73 to 0·89	Pharoah and colleagues [31]
Probabilities for further investigations and treatment (after screening or diagnosis)		
Hypertension		
Adherence to management	75% (in one-way SA: 50%)	Moran and colleagues [54]
Bowel cancer		
Screening positive and requiring further investigation	1·84 to 2·1%	Raine and colleagues [58]
Visit at specialist clinic for further investigation if screening was positive	74·7 to 91·3%	Logan and colleagues [59]
Bowel cancer if screening was positive	9·09 to 11·11%	As above
Pre-cancer polyps if screening was positive	24·48 to 29·92%	As above
Admission for bleeding due to further investigation	0·39 to 0·48%	Tappenden and colleagues [37]
Osteoporosis		
Prescription of drugs to person identified with osteoporosis	99·6%	Presentation by Shepstone at National Osteoporosis Conference 2016; [58] refers to findings from SCOOP study
Cataract		
Corrected with glasses if referred to eye exam	50 to 67%	Lennox and colleagues [51]
Surgery if referred to eye exam	5 to 9%	Expert view
Glaucoma		
Adherence to treatment	63·9 to 78·1%	Okeke and colleagues [61]; mean of 71%, value range +/− 10%
Hearing problems		
Hearing problem due to blocked ear wax (i.e. no referral required)	15·7 to 50%	Robertson and colleagues [20] and Clegg and colleagues [48]
Hearing problem not due to blocked ear wax and referral made to specialist	50 to 84·7%	Derived as residual from above
Person referred to specialist assessment attends it	80 to 90%	Expert view
Person assessed by specialist as requiring hearing aid	42·2 to 51·6%	Lennox and colleagues [50]
Person requiring hearing aid accepts and starts using it	36·8 to 86%	Morris and colleauges [59]
Breast cancer		
Breast cancer identified through mammogram	73·3 to 93·8%	Sinclair and colleagues [63]
Relative risk of over-diagnosis for women invited to mammography	1·19	As above

### Quality of life weights

Quality of life weights (utilities) were attached to health states, which included different progression states for some conditions. Data on health utilities were taken from national (economic) evaluations and referred to the general population in England [37, 38, 41, 44–47, 62–66]. For some health conditions (diabetes and cataract), present values of differences in QALY gains (or losses) linked to early identification or closer monitoring were already available in aggregated form from economic modelling studies, which included disease progression as relevant for the condition [41, 47]. For hearing impairment, only incremental values of health utility gain linked to ear-wax removal (a common problem in this population) and to hearing aid were available [61], and we thus assigned those to additional probabilities for people to benefit from AHC. Quality of life parameters, their values, sources and descriptions are shown in Table 5.

**Table 5:** Parameter values (deterministic, in ranges) for modelling: Quality of life weights (health utilities), ∆ health utilities and ∆ QALYs

	Data	Source and details
Health utilities (including ∆) and QALYs		
Stroke	0·31 to 0·94	Lovibond and colleagues [37]
Coronary heart disease	0·55 to 0·79	Derived from Lovibond and colleagues [37] and Dyer and colleagues [64]; weighted average of health utility values for heart failure (0.645), angina (0.77) and MI (0.76); weighted with their proportions of CHD (as above)
Hypertension (without cardiovascular event)	0·704 to 0·909	Lovibond and colleagues [37]; refers to general population health utilities from Health Survey England data
Bowel cancer	0·697 (+/−10%)	Whyte and colleagues [65]
Bowel cancer stages Duke’s A, B, C,D	0·74; 0·70; 0·5; 0·25 (+/− 10%)	Tappenden and colleagues [66]
Breast cancer	0·627 to 0·767	Whyte and colleagues [65]
HIP fracture, 1st year	0·64 to 0·77	
Without HIP fracture, 50 to 60 years	0·6 to 0·85	As above
Without HIP fracture, 60 years +	0·55 to 0·82	As above
Glaucoma, mild	0·72 to 0·88	Burr and colleagues [46]
Glaucoma, moderate	0·67 to 0·82	As above
Glaucoma, severe	0·64 to 0·78	As above
Health utility gain (∆) from removed ear wax	0·0054 to 0·0066	Morris and colleagues [62]
Health utility gain (∆) from hearing aid	0·035 to 0·105	NICE [41]
QALYs (∆), controlled vs. uncontrolled diabetes in non-overweight patients	−0·07 to 0·22	Clarke and colleagues [38]; refers to present value of QALYs gained for intensive vs. standard management
QALYs (∆), controlled vs. uncontrolled diabetes in overweight patients	−0·04 to 0·48	As above
QALY gain linked to cataract surgery	0·084 to 0·963	Frampton and colleagues [47]

## Results

Findings of the base case analysis are shown as averages per person (Table 6). AHCs led to a mean QALY gain of 0.074. The 95% Confidence interval (CI) ranged from 0.072 to 0.119. Mean incremental costs were £4787 with a CI 95% of 4773 to 5017. The mean ICER was £85,632 (95% CI 82,762 to 131,944). The yearly cost of an AHC was £258 per person. For health service decision-makers in England an ICER of less than £20,000 (or in some circumstances less than £30,000) is assumed to indicate the cost-effectiveness of an intervention [42]. Thus, AHC could not be considered cost-effective.

**Table 6:** Base-case analysis results (probabilistic) – cost effectiveness annual health checks (AHC) vs. standard care, all prices in £ 2015/16, per person

	Incremental costs	Incremental QALYs	Incremental cost-effectiveness ratio (ICER)
Mean	£4787.24	0·0743	£85,631.95
Standard deviation	£230.05	0·0456	£46,312.30
95% Confidence interval	£4772.98 to £5017.29	0·0715 to 0·119	£82,761.49.90 to £131,944.25

**Fig. 2:**
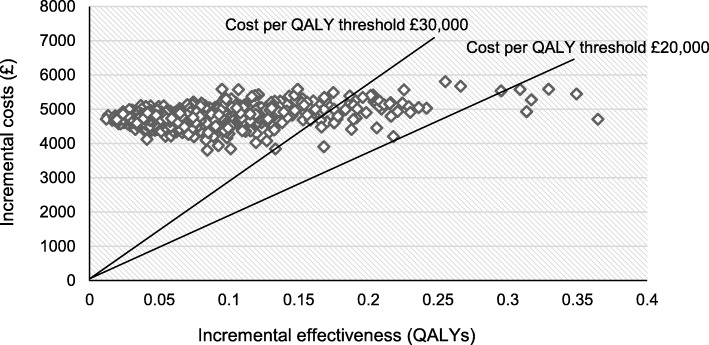
Probabilistic sensitivity results presented as scatter plot (cost-effectiveness plane), all prices in £ 2015/16

Another way of presenting our findings, including the uncertainty surrounding them, is through a cost-effectiveness plane (Fig. 2). The graph shows the incremental effects (measured in QALYs) on the x-axis and incremental costs on the y-axis. The dots represent the results of 1000 Monte Carlo simulations. As can be seen, only a few dots lie below the lines that represent the cost per QALY threshold for cost-effectiveness at £20,000. At a more generous threshold of £30,000 per QALY (which used to be and is still considered sometimes the upper range of a cost per QALY threshold), a few more dots lie under the line. However, the vast majority of dots are centred at incremental costs of £5000 and incremental QALY gains of less than 0.1, suggesting ICERs of above £50,000 and higher.

Findings from additional one-way sensitivity analysis showed that the cost of an AHC was the only parameter that substantially influenced the results and could turn the decision whether AHCs were cost-effective from a negative to a positive one. Findings in Table 7 show that if AHC could be provided at £50 per person per year then the probability of cost-effectiveness would be 70.1% (88.6%) at a cost per QALY threshold of £20,000 (£30,000); if the costs of annual health checks were £75 per person per year the probability would be 37.1% (66.1%); and at a cost of £100 per person per year they would no longer be cost-effective (that is, their probability of being cost-effective was less than 50%).

**Table 7:** One-way sensitivity analysis for different costs of annual health checks and impact on ICER and probability of cost-effectiveness, all prices in £ 2015/16, per person

Cost of the intervention	Mean ICER (95% CI)	Probability of cost-effectiveness at cost per QALY threshold of £20,000 (£30,000)
£50	£17,760 (£17,180 to £27,110)	70.1% (88.6%)
£75	£25,861 (£24,967 to £40,274)	37.1% (66.1%)
£100	£34,959 (£33,818 to £53,363)	25.5.9% (49.5%)
£150	£48,237 (£46,746 to £72,281)	7.1% (22.2%)

## Discussion

The aim of our study was to explore the likely cost-effectiveness of a strategy, in which AHC are incentivised for people with ID as they age versus a strategy in which standard care is provided. Modelling was used to address large evidence gaps in this area. This included utilising a wide range of data sets and consulting experts on parameters and values that informed the model. Findings from our study suggest that AHCs provided to older people with ID are unlikely to be cost-effective from a health service perspective. This conclusion was robust across many scenarios; the only scenario in which AHCs were cost-effective was when their annual cost was reduced to about one-third of their current cost (a figure estimated by experts in the field).

One strength of this study was that the potential cost-effectiveness of AHCs was examined using appropriate methods to take account of uncertainties [67]. Second, experts were consulted on the model structure, inclusion of health conditions and parameters that were uncertain. Third, conservative assumptions were made to reflect the realities of current practice in England. For example, the cost of an AHC included not only cost incurred by general practice but also the cost of additional support provided by support workers, which help people to: attend medical appointments; understand the nature and reasons of visits and tests and test results. Whilst not everyone will need this kind of support and support workers will be employed independently of whether they provide help with AHCs, it is a relevant opportunity cost since support workers’ time could be otherwise spent on helping the individual with something else or helping another person. Fourth, the analysis was informed by data from two recent, large-scale studies on this topic, helping to fill some evidence gaps (namely incidence of health conditions for this population [17] and on effectiveness of AHCs at the national level [23]).

This exploratory study has limitations linked to the many gaps in evidence, including those of: health utility values; incidence rates for some health conditions; access rates to secondary care treatment; and unit costs. Values for those parameters were taken from studies that referred to the general population; it is possible that they are different for people with ID. For example, there is currently not much knowledge about the validity of generic health-related quality of life measures such as the EQ-5D for this population, which is the measure used to derive health utilities [68, 69]. Whilst the experts we consulted thought that health utilities for this population were lower than in the general population due to the pervasive disadvantage and discrimination they experienced, no evidence could be identified to inform such values. However, this is unlikely to have influenced our incremental or net findings substantially since utilities would apply to all health states and would not change incremental QALYs, which are calculated in relative terms. A number of important health conditions could not be included in the modelling either because their incidence was not known from IDS-TILDA (e.g. asthma, chronic obstructive pulmonary disease) or because there was a lack of data on the costs and outcomes of earlier identification or treatment (e.g. thyroid problems, epilepsy, arthritis). Especially for conditions like dementia and mental illness, more evidence is needed about how to best identify those conditions in this population and about the (cost-) effectiveness of treatment and support options. In addition, for the majority of health conditions no evidence was available on follow-on treatments in secondary care once health conditions have been identified as a result of AHCs.

The impact of decent additional support in secondary care on access is currently not known. This also includes a lack of knowledge about the cost of this kind of additional support. In addition to those limitations, we were also unable to include a potential impact of earlier identification and treatment of health conditions on care home admission. Decisions about whether a person with ID is admitted to a care home are strongly influenced by their deteriorating health [70]. However, a lack of data prevented us from considering this potential economic impact. Similarly, we were not able to consider the impact on carers. This refers to the costs linked to the time spent by carers for supporting the person with ID ahead of, during and after medical appointments. This includes their traveling time to get to health care facilities. This cost is likely to present a large cost component of the overall support for people with ID. Whether this cost is influenced through the introduction of AHCs is currently not known, and presents an area where research is needed.

Despite those limitations, our study is the first analysis of long-term costs and outcomes of health checks for people with ID. The need for economic evidence in this area has been highlighted previously [10, 16, 23]. Previous studies which evaluated the cost-effectiveness of AHCs have been small-scale, short-term or included only a limited range of costs [16, 71, 72]. Findings from those studies suggest that there might be some improvements in overall health or reductions in service use but those impacts did not reach significance. None of the studies included the additional support from a support or social worker in their costs of AHCs; as a result their cost estimates for the intervention were substantially lower than ours, and authors were more likely to conclude that health checks were likely to be cost-effective. None of those studies looked at the population of older people specifically. From the perspective of health service expenditure alone, this is an important population given the high rates of health and social care service utilisation in old age [73].

As longevity improves for people with ID, it is imperative to consider how to best support the complex needs of this population in order to make the most cost-effective use of resources. Our findings highlight some of the dilemmas that commissioners and strategic decision-makers face. For example, changing only one part of the system (here: the identification of health conditions in primary care) is not leading to health-related quality of life improvements or reductions in mortality if people are not able to access and benefit from effective treatment options provided in other parts of the system (in particular in secondary care). In current practice, many hospitals fail to provide care that is consistently accessible to people with ID [74–77, 5]. Similarly, national screening programmes are not provided in a way that they are accessible for people with ID [78–80]. The need for collaborative approaches and wider system change in order to reduce health inequalities for this population has been highlighted in a number of studies [77–82].

Our findings should also be considered in the context of health checks provided to the general population of older people. Health checks have now been introduced in England for the *general* population aged 40 to 75 years. Concerns have been raised about their value for money, and their ability to achieve more benefits than harms has been questioned [83, 84]. Those concerns stem from: gaps in evidence concerning effective interventions and best practice when test results are positive; difficulties in explaining to patients the pros and cons of intervening versus not intervening during early disease stages; and the challenge of achieving behaviour change within one or two visits. “Although the goal is improved health outcomes, the pathway is long and tenuous, with attrition at each point along the way” [83]. Many of these challenges are likely to apply as much if not more to *older people with ID*.

Legislation and guidance request that barriers for people with ID in accessing health services are removed and reasonable adjustments are made [85–87]. Therefore, ethical considerations need to inform resource allocation decisions alongside economic ones. In current practice AHC offered to people with ID are a main policy vehicle for promoting a more equal access to health services. Thus, removing them might be considered highly unethical.

Seeking to respond to those challenges, the national guideline of which this study was a part made a number of recommendations about the provision of AHCs [25]: In particular:

- AHCs should be followed by prompt referrals to specialist services as needed; information on follow-on actions should be recorded;
- Practitioners carrying out AHCs should inform people about available health services including national screening programmes;

The guideline also recommends further research into the (cost-) effectiveness of alternative models and approaches for identifying health conditions and increasing access to treatment. This includes the role of well man and women clinics. In addition, the guideline makes a number of recommendations to improve accessibility of health services beyond the provision of AHCs. It sets out a system-wide responsibility to:

- Support people’s communication needs and information preferences; this might include: extending appointment times; contacting persons before appointments; reminding people of appointments; providing written information in an accessible format; using visual aids when explaining procedures or results; supporting the presence of an advocate or someone the person trusts at appointments;
- Increase peoples’ awareness of changing health needs due to ageing; this might include providing training for people and their family members in recognising and managing ageing related changes.

Furthermore, the guideline requests the introduction of new roles to make those changes happen. This includes a single lead practitioner as point of contact in each health care setting as well as champions in health care teams who develop specific knowledge and skills working with this population. Those recommendations are - while important and desirable - also ambitious in the current financial climate. The feasibility of their implementation in practice remains to be seen.

## Conclusions

This explorative study is the first to estimate the cost-effectiveness of health checks for people with intellectual disability as they age. It is also the first economic modelling study of health checks for people with intellectual disability at any age. The findings from this study suggest that the current focus by governments on providing yearly health checks for people with ID as they age might not be good value for money. Findings from this study informed recommendations in a guideline by the National Institute for Health and Care Excellence in England. Future research should investigate the impact of AHCs and alternative models of identifying health conditions on: the identification of health conditions such as dementia and mental health disorders; access to secondary care treatment and screening programmes; health-related quality of life improvements; impact on carers and support workers.

## Additional file

Additional file 1: Literature review(s). Details of how the literature was reviewed for this study. **Table S1.** Rational for including or excluding health conditions (detailed description with references). Summary of evidence for each health condition with conclusions about expected economic impact and feasibility for carrying out modelling. (DOCX 29 kb)

## Abbreviations

AHC: Annual health checks
CHD: Coronary heart disease
CI: Confidence interval
ICER: Incremental cost-effectiveness ratio
ID: Intellectual disability
IDS-TILDA: Intellectual Disabilities Supplement to the Irish Longitudinal Study on Aging
NICE: National Institute for Health and Care Excellence
PSA: Probabilistic sensitivity analysis
QALY: Quality adjusted life years
