# Supplementary material for: Access to health care for older people with intellectual disability: a modelling study to explore the cost-effectiveness of health checks
Source: BMC Public Health. 2019 Jun 7;19:706. doi: 10.1186/s12889-019-6912-0 (PMC6556058; doi:10.1186/s12889-019-6912-0)
Supplement: Supplementary file 1 — Literature review(s). Details of how the literature was reviewed for this study. Table S1. Rational for including or excluding health conditions (detailed description with references). Summary of evidence for each health condition with conclusions about expected economic impact and feasibility for carrying out modelling. (DOCX 29 kb) [file 12889_2019_6912_MOESM1_ESM.docx]

**Supplementary file 1**

**Literature review(s)**

Systematic searches for economic studies in this area were carried out in January 2016 (and update searches in February 2017) as part of the review for the national guideline, for which the work was carried. They informed the focus of this study. Searches were run across CINAHL, EMBASE, MEDLINE, PsychINFO, SSCI, NHS EED and EconLit, restricting the search to 10 years from 2005 to 2015 and with no language restrictions. Concepts of 1) older people, ageing, future planning, or aged care services and 2) intellectual or learning disabilities were translated into search strategies using subject heading and free text terms. No economic study was identified that specifically related to an ageing population.

We also carried out additional pragmatic searches and contacted leading researchers, who had published on health checks for people with learning disabilities to ensure we had not missed any relevant studies as part of the systematic searches. We identified n=3 studies which measured service use, costs or cost-effectiveness; all of them were based on small sample sizes and none of them was a modelling study.

Additional pragmatic literature searches were carried out to identify evidence on: prevalence and incidence of health conditions in this population; effectiveness of annual health checks in terms of identification or management of aging-relevant health conditions; effectiveness of early identification or enhanced management of health conditions in terms of health outcomes (in the form of health-related quality of life and mortality); costs of annual health checks; costs of identification and management (treatment) of health conditions (including cost differences of early versus late identification and treatment). Our searches were pragmatic and focused on studies that were from the UK and published in recognised sources that followed standardised methods (such as health technology assessments, NICE guidelines, Cochrane systematic reviews and meta-analysis). Searches were done using citation mining. In addition, leading researchers in this field were contacted to refer us to studies in this field This included: Dr Laurence Taggart, Reader in School of Nursing at Ulster University; Dr Andre Strydom, Senior Lecturer in the Mental Health Sciences Unit at University College London; Dr Renee Romeo, Senior Lecturer in Health Economics at King’s College London; Prof Christopher Hatton, Professor of Psychology, Health and Social care at the Centre for Disability Research at University of Lancaster; Prof Martin Knapp, Professor of Social Policy and Director of Personal Social Services Research Unit (PSSRU) at London School of Economics and Political Science.

**Supplementary table 1**

**Rational for including or excluding health conditions (detailed descriptions with references)**

| **Health condition** | **Included/ excluded** | **Rationale** |
| --- | --- | --- |
| Arthritis | Excluded | Prevalence was found to be 17% in IDS-TILDA. There was no evidence that annual health checks change identification rates. There were different types of arthritis and with no standard treatment currently recommended by the European Society for Clinical and Economic Aspects of Osteoporosis and Osteoarthritis (ESCEO)^36^ the Committee agreed that there was a need to define a reference case for osteoarthritis and achieve consensus on what constitutes ‘standard optimal care’. Despite high prevalence, expected impact on costs and outcomes was likely to be only low or medium due to uncertainties around identification and management. Arthritis was thus excluded from the modelling. |
| Blood pressure (identification and management) | Included | There is a high prevalence of objectively measured blood pressure – 18.1% in IDS-TILDA.^3^ People in the annual health check group were more likely to be identified with hypertension (= high blood pressure) and more likely to receive blood pressure management.^18,19^ The expected impact on costs and outcomes was high: high blood pressure is a major risk factor for life-threatening and costly conditions including coronary heart disease (CHD) and stroke (see for example, ^20^); there is strong (cost-) effectiveness evidence for blood pressure management which can reduce the risk of developing long-term conditions, in particular stroke and heart disease; and robust evidence shows that treating blood pressure in older adults reduces stroke, cardiac events and mortality.^21^ |
| Body mass index, cholesterol, weight | Excluded | Prevalence of overweight is as high as 42.5% (this referred to a subsample who engage in height and weight measurement). Health promotion in the form of advice on nutrition and physical exercise is an important area for action but the role of health checks in improving health promotion is not clear and there is a lack of evidence on (cost-)effectiveness of health promotion interventions.^25^ Generally, research suggests that it is difficult to achieve health promotion targets (such as a healthy diet to reduce obesity which is more common) among people with ID^24^ although there is some evidence that education about exercise and nutrition might lead to changes in health attitudes.^26^ Overall, the evidence that health checks can influence costs or outcomes in health promotion was considered too weak for this topic to be included in the modelling. |
| Bowel cancer screening | Included | Bowel (= colorectal) cancer is a common cancer and represents 11.3% of all cancers in the general population;^1^ furthermore, it is the second leading cause of cancer death in the general population.^9^ Prevalence is not known for people with ID but death rates from bowel cancer have been found to be higher in this population.^10^ RCTs have shown that screening for bowel cancer (using the fecal occult blood test – FOBt) can reduce mortality by 16% in people offered screening and up to 25% in those accepting it; ^11,12,13^ it is proven to be highly cost-effective in different high-income countries including the UK.^14,15^ An evaluation of the national Bowel Cancer Screening Programme in England showed that the uptake of FOBt in the general population ranged from 40 to 60%.^12^ Generally, low uptake is a major public health concern especially among certain populations such as those at socioeconomic disadvantage, ethnic minorities and people with an ID.^16^ Expected impact on costs and outcomes was high due to the high prevalence, the availability of a national screening programme, the deathly nature of the condition and the evidence of (cost-) effectiveness of treatment. |
| Breast cancer (screening via mammogram) | Included | Breast cancer is a common cancer, representing 15% of all cancers.^1^ Rates are expected to be the same or higher for older people with ID compared with the general population. ^4,5,6^ Despite the national NHS Breast Cancer Screening programme, uptake of mammograms is a particular issue in this population with rates in uptake being much lower (50%) than in the general population (80%; ^3,6^ ). It has been suggested that there are many missed opportunities in primary care for proving reminders and better information about screening. ^4,7,8^ The many barriers to breast screening as well as the importance of general practice staff in reminding women and their carers opportunistically about breast cancer screening have been suggested in the literature. ^8^ The expected impact on costs and outcomes is high due to the evidence that screening of this type of cancer is feasible and cost-effective (hence the national screening programme) and evidence that additional information provided by general practitioners can increase uptake. |
| Cataract | Included | The prevalence of cataract was 19% in this population in IDS-TILDA. ^3^ This is much higher than the prevalence found in the general population of 10%.^3^ Evidence shows that vision problems such as cataract often remain unrecognised without health checks.^28^ Evaluations of annual health checks for people with ID consistently found that people in the health check group were more likely to get eye exams.^18,28,29^ The expected impact on costs and outcomes was medium to high: although difficult to quantify, the impact of vision impairments was expected to be larger than for the general population, reducing substantially people’s ability to communicate, carry out practical tasks and be socially active. Cataract is thought to contribute to lower quality of life, social isolation, loss of independence and physical health problems.^28^ Cataract was thus included in the modelling. |
| Cervical cancer screening | Excluded | While it is important to offer smear tests to women and provide information about the pros and cons of screening for cervical cancer (and to carry out smear tests), prevalence of cervical cancer in this population is very low (in particular due to much lower sexual activity than in the general population) and the expected impact on costs and outcomes was thus considered to be low. For this reason, cervical cancer screening was not included in the modelling. |
| Chronic obstructive pulmonary disease (COPD) and asthma | Excluded | Prevalence data on asthma were not established in IDS-TILDA. There was a lack of evidence that health checks led to differences in identification and management of asthma or COPD (for example, a systematic review by Robertson et al. 2014 did not mention asthma or COPD). It was therefore excluded from the modelling. |
| Dementia | Excluded | Prevalence of dementia is much higher than in the general population, in particular for people with Down’s syndrome. In IDS-TILDA the prevalence was 30% in people with Down’s syndrome (dementia was not measured for people without Down’s syndrome). Onset of dementia can be earlier than in the general population, in particular for people with Down’s syndrome. NICE^46^ recommends that dementia should be assessed, however, there are many uncertainties concerning what effective identification should look like and who should be doing it. Evidence on effectiveness of interventions for adults with ID might not necessarily apply to older people, in particular those with dementia. For example, evidence for people with dementia from recent health technology assessment carried out by Banarjee et al.^50^ found that anti-depressants are not effective in reducing depression for people with dementia. Leong et al.^51^ found that evidence is at best inconclusive; Power et al.^52^ found no preventative effect of statins on dementia. It was thus not possible to include dementia in the model. |
| Epilepsy | Excluded | The nature of epilepsy can change for older people, and people can also develop new epilepsy in older age.^3,40^ NICE recommends the same therapies and interventions for older people as for other ages, but possibly lower doses of anti-epileptic drugs.^41-43^ There was some evidence that drugs could help older people to become seizure-free at least for a certain period.^41-43^ The impact on costs and outcomes was expected to be large, considering that that 30% of acute seizures (those with status epilepticus) carried a mortality of 40%.^41-43^ However, data were from dated, small studies and could not be used to inform the modelling. |
| Heart disease and stroke | Included (indirectly) | Hypertension and diabetes have been found to be predictors of stroke and heart disease.^27^ The issue of strong overlap between these conditions means that modelling each of them separately was not appropriate as they are on the same pathway for the majority of people. These conditions were thus addressed indirectly through the modelling work for diabetes and hypertension. |
| Hearing impairment | Included | Prevalence has been found to be at least 40%.^30^ A study found that most people have not had their hearing tested.^31^ Evaluations of annual health checks for people with ID found that people in the health check group were more likely to get earwax removed, hearing assessments and hearing aids than people in the standard care group.^18,28,29^ There are expected high impacts in regard to costs and outcomes: evidence shows that the cost impact hearing impairment in the general population of older people is very large ^32^ and the substantial health gains of earwax removal and hearing aids.^31^ Although difficult to measure in quantitative terms, wider benefits such as a reduction in social isolation and an increase in mobility, along with physical health improvements are expected to be larger than in the general population.^33^ |
| Glaucoma | Included | Prevalence of glaucoma was 2.7% in IDS-TILDA.^3^ Evidence from evaluations of annual health checks for people with ID consistently shows that people in health check groups are more likely to get eye exams.^18^ Expected impact on costs and outcomes was high: although difficult to quantify, the impact of vision impairments is expected to be larger than for the general population, reducing more substantially their abilities to communicate, carry out practical tasks and be socially active. Vision impairments can contribute substantially to lower quality of life, social isolation, independence and physical health problems.^28^ They can often be relatively easy treated and promise cost-effectiveness. They were thus included the modelling. |
| Immunisation status | Excluded | IDS-TILDA found that vaccination levels to prevent influenza were over 90%. Older people with ID are targeted by a national screening programme and general practices are incentivised through other means. This suggested a more limited role of health checks in further improving uptake. |
| Lung cancer/ smoking | Excluded | Not much is known about the prevalence of lung cancer in this population; in the general population, 12.7% of all cancers are lung cancer, and it is likely that rates are similar for this population. There is no national screening programme since no test has been found to be sufficiently robust in detecting lung cancer. Generally, early identification has been found to be difficult. Interventions on smoking cessation for people with ID lack theoretical frameworks and clear outcome measures.^17^ |
| Mental health | Excluded | In IDS-TILDA nearly 60% of older people with ID had received a diagnosis of a mental health condition,^44^ which is even higher than previous estimates in the adult population of 40%.^45^ Anxiety was the most prevalent (39.2%) followed by depression (26.7%) and manic depression (4.7%). A recently published NICE guideline recommends that mental health needs should be reviewed in annual checks alongside physical health needs.^46^ Health checks include questions about behaviour changes which can help to understand mental health needs. Evidence suggests that in practice people with ID commonly get the wrong diagnosis and are over-prescribed drugs in primary care.^47^ The majority of evaluations of health checks did not report on mental health (for example, the systematic review by Robertson et al.^28^ did not mention mental health problems), suggesting that a focus on mental health as part of health checks is still relatively new. In addition, the few feasibility studies available on evidence on (cost-) effectiveness of interventions refer to the general adult population and general evidence on effective treatment is inconclusive.^48,49^ Altogether there was not sufficient evidence to inform modelling in this area. |
| Osteoporosis (screening) | Included | IDS-TILDA found a prevalence of 16.4% for older people with ID, which is higher than the prevalence found in the general population of 14.3%.^3^ Srikanth et al. ^37^ found a very high prevalence of risk factors in this population, with 64% being on anti-epileptics, 23% being immobile and 20% (11%) having had falls (fractures). Findings from IDS-TILDA suggest that prevalence of falls might be up to 32% in women. Women are at particular risk of fracture following menopause (with 1 in 2 women having a fracture) and women with ID experience menopause earlier, which suggest that earlier identification in this group is particularly important. They are also less likely to report symptoms.^38^ The disease burden of osteoporosis is particularly high and 20% of older people die within a year of a fracture.^39^ There is evidence that treatment can reduce the risk of fractures and even mortality.^39^ Older people with ID are less likely to understand and report symptoms, and as a result do not get the appropriate treatment.^37^ While there is currently no national screening, NICE recommends risk assessment with tools like the FRAX in high-risk groups.^37^ The expected impact on costs and outcomes was considered medium to high due the high prevalence and the availability of screening tools that can lead to identification and the potential reduction in costly fractures. However, treatment persistence has also been found to be an issue, even in the general population, so this might reduce some of the potentially large positive impacts on fracture risk. |
| Prostate cancer | Excluded | Prevalence was not well known for this population but likely to be high; in the general population, prostate cancer is one of the most common cancer types in male older people (12.8% of all cancers). ^1^ However, there is currently no screening programme for prostate cancer because of the lack of a reliable test (current test is for Prostate Specific Agent, PSA). Side effects for overtreatment are substantial and the current conclusion in clinical guidelines is that the benefits do not outweigh the risks. There is a debate about treatment for people with elevated PSA levels. Possible strategies include MRS/MRI sequences. Cost-effectiveness evidence from recent health technology assessment ^2^ shows a high degree of uncertainty surrounding key parameters for any such strategy and so no clear recommendation was made. Whilst it is important to offer PSA tests to older men with ID as part of annual health checks (currently about 60% of people with ID get checked for prostate according to IDS-TILDA) ^3^ but agreed that modelling was difficult to the uncertainty in the data about prevalence and best treatment options. |
| Thyroid problems | Excluded | The prevalence of thyroid problems was 21% in IDS-TILDA.^3^ Evidence shows that health checks lead to an increased number of thyroid function tests. This refers specifically to hypothyroidism, which is particularly common for people with Down’s syndrome. The rate of hypothyroidism in people with ID is 2.9 times that in the general population.^34^ Regular (annual) monitoring is already incentivised by a quality and outcome framework. National data suggest that up to 96% of patients with hypothyroidism had received thyroid function tests in 2010/11. Current guidelines suggest that people with Down’s syndrome should be screened annually for hypothyroidism. The Committee agreed that thyroid conditions should be checked for all older people with ID, not just people with Down’s syndrome. Overt hypothyroidism is linked to quality of life impairments and can lead to mortality. However, while diagnosis is relatively straightforward, treatment in older people is complex and does not necessarily lead to improved health-related quality of life in practice: up to half the population on thyroid replacement therapy were being over- or undertreated and there are substantial side effects of treatment. Evidence suggests that despite regular monitoring the dose often remains unchanged when people age, and 20% of older people are over-treated, which increases the risk of fractures.^35^ The most common drug used for thyroid replacement therapy is levothyroxine, which is a low cost drug, so that the impact on costs is likely to be small. There is an overall lack of evidence suggesting that expected impact of identification or monitoring through annual health checks is likely to have a large impact on costs or health outcomes. |

References

[1] Cancer Research UK (2014) The twenty most common cancers 2014, number of new cases UK, http://www.cancerresearchuk.org/sites/default/files/cstream-node/inc_20common_persons_I14.pdf, lst accessed 17 May 2017.

[2] Mowatt G, Scotland G, Boachie C et al. (2013) The diagnostic accuracy and cost-effectiveness of magnetic resonance spectroscopy and enhanced magnetic resonance imaging techniques in aiding the localisation of prostate abnormalities for biopsy: a systematic review and economic evaluation. Health Technol Assess 17(20).

[3] McCarron M, Buke E, Clearly E, Carroll R, McGlincey E, McCallion P (2014) Changes in Physical and Behavioural Health of with an Intellectual Disabilities Ageing, in E Burke, P McCallion, M McCarron (eds) Advancing years, different challenges: wave 2 IDS-TILDA, findings on the ageing of people with and intellectual disabilities. Dublin: School of Nursing and Midwifery, the University of Dublin, Trinity College.

[4] Davies N, Duff M (2001) Breast screening for older women with intellectual disabilities living in community group homes. Journal of Intellectual Disabilities Research 45(3): 253–7.

[5] Hogg J, Tuffrey-Wijne I (2008), Cancer and intellectual disabilities: a review of some key contextual issues. Journal of Applied Research in Intellectual Disabilities 21(6): 509–18.

[6] Truesdale-Kennedy M, Taggart L, McIlfatrick S (2011) Breast cancer knowledge among women with intellectual disabilities and their experiences of receiving breast mammography. Journal of Advanced Nursing 67(6): 1294–304.

[7] Glover G, Ayub M (2010) How people die with ID, improving health and lives. London: ID Observatory, Department of Health.

[8] Wilkinson JE, Lauer E, Freud KM, Rosen AK (2011) Determinants of mammography in women with intellectual disabilities. J Am Board Fam Med 24(6): 693–703.

[9] Macafee, D. A., Waller, M., Whynes, D. K., Moss, S., & Scholefield, J. H. (2008). Population screening for colorectal cancer: the implications of an ageing population. Br J Cancer, 99(12), 1991-2000.

[10] Glover G, Williams R, Heslop P, Oyinola J, Grey J (2016) Mortality in people with intellectual disabilities in England. Journal of Intellectual Disabilities Research, 61: 62-74.

[11] Hewitson P, Glasziou P, Watson E, et al. (2008) Cochrane systematic review of colorectal cancer screening using the fecal occult blood test (hemoccult): an update. Am J Gastroenterol 103: n1541e9.

[12] Logan RF, Patnick J, Nickerson C et al. and English Bowel Cancer Screening Evaluation (2012) Outcomes of the Bowel Cancer Screening Programme (BCSP) in England after the first 1 million tests. Gut, 61(10): 1439–46.

[13] Towler B, Irwig L, Glasziou P et al. (1998) A systematic review of the effects of screening for colorectal cancer using the faecal occult blood test, hemoccult. BMJ 317: 559e65.

[14] Lansdorp-Vogelaar I, Knudsen AB, Brenner H (2011) Cost-effectiveness of colorectal cancer screening. Epidemiol Rev 33: 88–100.

[15] Tappenden P, Chilcott J, Eggington S, Patnick J, Sakai H, Karnon J (2007) Option appraisal of population-based colorectal cancer screening programmes in England. Gut 56(5): 677–84.

[16] NDTI (2013) Improving the uptake of screening services by people with ID across the South West Peninsula – a strategy and toolkit, November 2013. Bristol: National Development Team for Inclusion, Norah Fry Research Centre.

[17] Kerr, S, Lawrence, M., Darbyshire, C., Middleton, A. R., & Fitzsimmons, L. (2013). Tobacco and alcohol-related interventions for people with mild/moderate intellectual disabilities: a systematic review of the literature. Journal of Intellectual Disability Research, 57(5), 393-408.

[18] Buszewicz M, Welch C, Horsfall L et al. (2014) Assessment of an incentivised scheme to provide annual health checks in primary care for adults with intellectual disabilities: a longitudinal cohort study. Lancet Psychiatry 1: 522–30.

[19] Cooper SA, Morrison J, Allan LM, et al. (2014) Practice nurse health checks for adults with intellectual disabilities: a cluster-design, randomised controlled trial. Lancet Psychiatry 1: 511–21.

[20] Kannel WB (2009) Hypertension: reflections on risks and prognostication. Med Clin North Am 93(3): 541–58.

[21] Weiss J, Kerfoot A, Freeman M et al. (2015) Benefits and harms of treating blood pressure in older adults: a systematic review and meta-analysis. VA ESP Project #05-225; 2015.

[22] McVilly, K., McGillivray, J., Curtis, A., Lehmann, J., Morrish, L., & Speight, J. (2014). Diabetes in people with an intellectual disability: a systematic review of prevalence, incidence and impact. Diabet Med, 31(8), 897-904.

[23] MacRae S, Brown M, Karatzias T et al. (2015) Diabetes in people with intellectual disabilities: a systematic review of the literature. Research in Developmental Disabilities 47: 352–74.

[24] Taggart L, Coates M, Truesdale-Kennedy M (2013) Management and quality indicators of diabetes mellitus in people with intellectual disabilities. Journal of Intellectual Disabilities Research 57(12): 1152–6.

[25] Brooker K, Van Dooren K, McPherson L, Lennox N, Ware R (2015) A systematic review of interventions aiming to improve involvement in physical activity among adults with intellectual disabilities. J Phys Act Health 12(3): 434–44.

[26] Heller T, Sorenson A (2013) Promoting healthy ageing in adults with developmental disabilities. Dev Disabil Res Rev 18(1): 22–30.

[27] Morrissey EC, Durand H, Nieuwlaat R et al. (2016). Effectiveness and content analysis of interventions to enhance medication adherence in hypertension: a systematic review and meta-analysis protocol. Syst Rev 5(1): 96.

[28] Robertson J, Hatton C, Emerson E, Baines S (2014) The impact of health checks for people with intellectual disabilities: an updated systematic review. Research in Developmental Disabilities 35: 2450–62.

[29] Lennox N, Bain C, Rey-Conde T, Purdie D, Bush R, Pandeya N (2007) Effects of a comprehensive health assessment programme for Australian adults with intellectual disabilities: a cluster randomized trial. Int J Epidemiol 36(1): 139–46.

[30] Bent S, McShea L, Brennan S (2015) The importance of hearing: a review of the literature on hearing loss for older people with ID. British Journal of ID 43(4): 277–84.

[31] Hardy S, Woodward P, Woolard P, Tait T (2011) Meeting the health needs of people with ID. RCN guidance for nursing staff, 2nd edn. London: Royal College of Nursing 61.

[32] Shield B (2006) Evaluation of the social and economic costs of hearing impairment. A report for hear-it. London: London South Bank University

Sinclair N, Littenberg B, Geller B, Muss H (2011) Accuracy of screening mammography in older women. AJR Am J Roentgenol. 197(5), 1268-1273.

[33] Felce D, Baxter H, Lowe K, Dunstan F et al. (2008) The impact of repeated health checks for adults with intellectual disabilities. Journal of Applied Research in Intellectual Disabilities 21: 585–96.

[34] NHS Digital (2016) Health and care of people with ID 2014–15, published 9 December 2016, ISBN 978-1-78386-893-3, http://www.content.digital.nhs.uk/catalogue/PUB22607/Health-care-learning-disabilities-2014-15-summary.pdf, Last accessed 14 Sept 2017.

[35] Turner MR, Camacho X, Fischer HD et al. (2011) Levothyroxine dose and risk of fractures in older adults: nested case-control study. BMJ 342: d2238.

[36] European Society for Clinical and Economic Aspects of Osteoporosis and Osteoarthritis (ESCEO);

[37] Srikanth R, Cassidy G, Joiner C, Teeluckdharry S (2011) Osteoporosis in people with intellectual disabilities: a review and a brief study of risk factors for osteoporosis in a community sample of people with intellectual disabilities. J Intellect Disabil Res 55(1): 53–62.

[38] Martin DM, Kakumani S, Martin MS, Cassidy G (2003) ID and the menopause. J Br Menopause Soc 9(1): 22–6.

[39] Zethraeus N, Borgstrom F, Strom O, Kanis JA, Jonsson B (2007) Cost-effectiveness of the treatment and prevention of osteoporosis – a review of the literature and a reference model. Osteoporos Int 18(1): 9–23.

[40] Burke E, McCallion P, McCarron M (2014) Advancing years, different challenges: wave 2 ids-tilda, findings on the ageing of people with and intellectual disabilities. Dublin: School of Nursing and Midwifery, the University of Dublin Trinity College.

[41] NICE (2018). Epilepsies: diagnosis and management. NICE guideline CG137, April 2018. London: National Institute for Health and Care Excellence.

[42] Stephen L, Brodie M (2000) Epilepsy in elderly people. Lancet 355(9213): 1441–6.

[43] Brodie MJ, Kwan P (2005) Epilepsy in elderly people. BMJ 331(7528): 1317–22, doi:10.1136/bmj.331.7528.1317.

[44] Mulryan N, Clearly E, McCallion P, McCarron (2014) Mental health, wellbeing and cognitive function in older adults with an intellectual disabilities in E Burke, P McCallion P, McCarron M (eds), Advancing years, different challenges: Wave 2 IDS-TLDA, findings on the ageing of people with and intellectual disabilities. Dublin: School of Nursing and Midwifery, the University of Dublin Trinity College.

[45] Cooper SA, Smiley E, Morrison J, Williamson A, Allan L (2007) Mental ill-health in adults with intellectual disabilities: prevalence and associated factors. The British Journal of Psychiatry 190(1): 27–35.

[46] NICE (2016). Mental health problems in people with ID: prevention, assessment and management. NICE guideline NG54, September 2016. London: National Institute for Health and Social Care.

[47] Glover G, Williams R, Branford D et al (2015) Prescribing of psychotropic drugs to people with ID and/or autism by general practitioners in England. Technical Report. Public Health England, London.

[48] Bouras N, Holt G (2004) Mental health services for adults with ID. The British Journal of Psychiatry 184(4): 291–2, doi:10.1192/bjp.184.4.291.

[49] Jahoda A, Melville CA, Per, C et al. (2015) A feasibility study of behavioural activation for depressive symptoms in adults with intellectual disabilities. Journal of Intellectual Disabilities Research 59(11): 1010–21

[50] Banerjee S, Hellier J, Romeo R, Dewey M, Knapp M, Ballard C, Burns A (2013) Study of the use of antidepressants for depression in dementia: the HTA-SADD trial – a multicentre, randomised, double-blind, placebo-controlled trial of the clinical effectiveness and cost-effectiveness of sertraline and mirtazapine. Health Technol Assess 17(7): 1–166.

[51] Leong, C. (2014). Antidepressants for depression in patients with dementia: a review of the literature. Consult Pharm, 29(4), 254-263.

[52] Power MC, Weuve J, Sharrett AR et al. (2015) Statins, cognition, and dementia – systematic review and methodological commentary. Nat Rev Neurol 11(4): 220–9.
